# Social value of a nutritional counselling and support program for breastfeeding in urban poor settings, Nairobi

**DOI:** 10.1186/s12889-018-5334-8

**Published:** 2018-04-02

**Authors:** Sophie Goudet, Paula L. Griffiths, Caroline W. Wainaina, Teresia N. Macharia, Frederick M. Wekesah, Milka Wanjohi, Peter Muriuki, Elizabeth Kimani-Murage

**Affiliations:** 1 Loughborough University School of Sport Exercise and Health Sciences, Loughborough, UK; 2 African Population and Health Research Center, Nairobi, Kenya

**Keywords:** Social return on investment, Exclusive breastfeeding, Community health volunteers, Intervention, Urban poor, Nairobi, Kenya

## Abstract

**Background:**

In Kenya, poor maternal nutrition, suboptimal infant and young child feeding practices and high levels of malnutrition have been shown among the urban poor. An intervention aimed at promoting optimal maternal infant and young child nutrition (MIYCN) practices in urban poor settings in Nairobi, Kenya was implemented. The intervention involved home-based counselling of pregnant and breastfeeding women and mothers of young children by community health volunteers (CHVs) on optimal MIYCN practices. This study assesses the social impact of the intervention using a Social Return on Investment (SROI) approach.

**Methods:**

Data collection was based on SROI methods and used a mixed methods approach (focus group discussions, key informant interviews, in-depth interviews, quantitative stakeholder surveys, and revealed preference approach for outcomes using value games).

**Results:**

The SROI analysis revealed that the MIYCN intervention was assessed to be highly effective and created social value, particularly for mothers and their children. Positive changes that participants experienced included mothers being more confident in child care and children and mothers being healthier. Overall, the intervention had a negative social impact on daycare centers and on health care providers, by putting too much pressure on them to provide care without providing extra support. The study calculated that, after accounting for discounting factors, the input ($USD 419,716) generated **$**USD 8 million of social value at the end of the project. The net present value created by the project was estimated at $USD 29.5 million. $USD 1 invested in the project was estimated to bring USD$ 71 (sensitivity analysis: USD$ 34–136) of social value for the stakeholders.

**Conclusion:**

The MIYCN intervention showed an important social impact in which mothers and children benefited the most. The intervention resulted in better perceived health of mothers and children and increased confidence of mothers to provide care for their children, while it resulted in negative impacts for day care center owners and health care providers.

**Electronic supplementary material:**

The online version of this article (10.1186/s12889-018-5334-8) contains supplementary material, which is available to authorized users.

## Background

In Kenya, sub-optimal maternal, infant and young child nutrition practices are documented, with consequent high levels of child malnutrition. The levels of malnutrition have gone down, for stunting from 35% in 2008 to 26% in 2014 [1]. Likewise in poor urban areas, inappropriate maternal nutrition, suboptimal infant and young child feeding practices and high levels of undernutrition have been shown while exclusive breastfeeding for six months is almost non-existent [2, 3]. This means that most children are failing to meet their nutritional requirements for optimal growth during infancy, and the development and health based on the WHO recommendation to exclusive breastfeeding in the first six months of a child’s life. Complementary feeding practices are suboptimal, especially with regard to the nutrient density of the foods fed to children under two years of age [3] and hence, fail to meet the nutritional requirement of growing infants. Poor breastfeeding and complementary feeding practices are immediate causes of undernutrition. In this poor urban setting in Nairobi, stunting, a form of undernutrition, stands at between 47% and 50% [4, 5]. Stunting is a major risk factor for morbidity and mortality and is also associated with adverse outcomes including compromised cognitive development, scholarly achievement and future economic productivity.

Interventions promoting optimal breastfeeding can reduce morbidity and mortality in children [6]. The baby friendly community initiative (BFCI) is a multifaceted program for promotion of optimal breastfeeding and infant and young child nutrition, and other practices including maternal nutrition in the community. BFCI is a complementary program to the Baby-Friendly Hospital Initiative developed by the World Health Organization and the United Nations Children’s Fund (UNICEF), with the aim of promoting breastfeeding in maternity facilities worldwide, and adopted in Kenya [7]. Given that many births (close to 40%) take place at home in Kenya [1], and also recognizing the need for continuum of care at the community even for those who deliver in hospital, the Ministry of Health has proposed the adoption of BFCI to bring breastfeeding counselling and support to the community as outlined in the country’s 2012–2017 Nutrition Action Plan (https://scalingupnutrition.org/wp-content/uploads/2013/10/Kenya-National-Nutrition-Action-Plan-2012-2017-final.pdf). The BFCI package (unpublished) adapted for implementation in Kenya involves an 8-step plan (the 8 steps are included in the Additional file 1: Table S1). BFCI relies on a network of community health volunteers (CHVs) to reach mothers in their homes. In Kenya, CHVs are part of the Community Health Strategy, a government initiative that aims at using CHVs to promote health in the community.

The African Population and Health Research Center (APHRC) with the support of the Ministry of Health in Kenya implemented a pilot project of the BFCI called Maternal Infant and Young Child Nutrition (MIYCN) project to assess effectiveness of the community health volunteer element of the program to inform implementation of the BFCI in Kenya. The intervention aimed to improve breastfeeding and other infant feeding practices, and consequently nutritional and health outcomes of children in urban poor settings in Nairobi. The pilot study, employing a cluster-randomized study design, was conducted in two slums where 14 community units (defined by the Government’s health care system) formed the unit of randomization (http://aphrc.org/wp-content/uploads/2016/03/FINAL-FILE-Design-draft-4-Social-return-on-investments-evaluation-report-31st-Mar-2016.pdf). A total of 1100 pregnant women and their respective babies were recruited and randomly allocated into the intervention and control groups and followed up. The community was mobilized and promotion on the proposed intervention was organized within the communities. CHVs were trained on standard care, maternal nutrition, breastfeeding and complementary feeding; deployed, supervised and incentivized. The mothers received regular, personalized, home-based counselling by trained CHVs on breastfeeding and complementary feeding and encouraged to comply for antenatal and postnatal (ANC/PNC) visits, birth planning, immunization, water and sanitation and hygiene (WASH) practices in relation to the child (the content of the counselling messages are in the Additional file 1: Table S2). At national to county level, policy and decision makers were engaged via stakeholder meetings. Regular assessment of knowledge, attitudes and practices on MIYCN was done, coupled with assessments of nutritional status of the mother-child pairs and diarrhea morbidity for their children. Children with severe acute malnutrition (SAM) were referred to therapeutic feeding centers. Sick children were referred to health facilities in the community. These children had increased home visits by CHVs. As opposed to the intervention group, the control group was not counseled by CHVs trained on MIYCN but those trained on standard care only, with distribution of standard MIYCN leaflets. The RCT findings showed that the MIYCN potentially had an impact on exclusive breastfeeding and stunting. The rate of exclusive breastfeeding for six months increased from about 2% at baseline (before the intervention) to approximately 55% for both intervention and control group [8]. The prevalence of stunting for children aged 6–12 months reduced from about 33% at baseline to about 30% in the intervention group, but increased to 38% in the control group. The lack of significant difference in exclusive breastfeeding rates between the two groups was considered to be due to potential contamination between them [8]. It is possible that the CHVs in the control group may have obtained knowledge on MIYCN from the standard training given to them (as it also includes messages on exclusive breastfeeding) or from other sources e.g. other NGOs and from the CHVs in the intervention group with whom they were interacting with. More MIYCN knowledge may have been obtained from the information materials that were provided to the CHVs in both arms.

The analysis presented here using a Social Return on Investment (SROI) approach aimed to quantify the social value created by the MIYCN intervention. [9]. In this paper, stakeholders; i.e. people who have been impacted by the intervention, were central in assessing and valuing the impact of the intervention.

## Methods

The methods used were based upon the SROI principles and steps as presented in the SROI guide [10] and in the practical guide for international cooperation [9]. The methodology used is described in the paper by Goudet et al. 2016 [10] and thus is not explained here. Data collection used a mixed methods approach (qualitatively using focus group discussions, key informant interviews, in-depth interviews, and quantitative stakeholder surveys, and revealed preference approach using value games [11]).

The stakeholders included individuals or organizations that were directly or indirectly impacted by the MIYCN intervention and as a result experienced a change that matters socially or economically. The research team elicited a potential stakeholder list. During the study’s inception meeting held in June 2015 in Nairobi, representatives from the Ministry of Health (at the county and national levels), UNICEF, USAID, Non-Governmental Organizations, research organizations and academic institutions contributed to firming up the identification of stakeholders. An influence and importance matrix was used to identify and select stakeholders (Additional file 1). Mothers who participated in the intervention, their children, the fathers, the grandmothers, the community health volunteers, the health care centers, the data collectors and the day care centers were identified(the list of stakeholders are included in the Additional file 1: Table S4). Children were too young to be interviewed thus the researchers relied on mothers to report their outcomes. Some identified stakeholders (grandfathers, other relatives, neighbors and media) were excluded from the data collection by the research team as the material impact as a result of the activities was assessed to be minor by the research team during the ‘identification of stakeholders’ phase. Some stakeholders were included in the data collection (shop owners, traditional birth attendants) but excluded in the final analysis by the research team as the impact on them was not tangible based on their responses. For example one shopkeeper kept mentioning how she had been advised on the use of the aqua tabs (tablets for sterilizing water) and how his business had increased as a result of stocking the aqua tabs and people buying. The MIYCN project did not counsel mothers on sterilizing water using aqua tabs but general hygiene including washing hands, boiling water for drinking.

### Qualitative component

The main objective of the qualitative work was to gain a general understanding of changes observed or experienced as a result of the intervention. The qualitative approach explored the impact of the intervention per stakeholder using data from eight focus group discussions (FGDs), 15 key informant interviews (KIIs) and 14 in-depth interviews (IDIs) involving 161 participants. Qualitative data collection was done through In-depth interviews (20) Key Informant Interviews (28) and Focus Group Discussions (19) with the potential stakeholders. A total of 162 participants, representing each stakeholder group were selected purposively from the study community to participate in the qualitative interviews. The selection process took into account their religion, ethnicity and village of residence to minimise bias. A pilot activity was conducted to pre-test the question guide and feedback from the pilot was used to review tools accordingly. The teams were trained on the qualitative tools during the 7 days trainings on the SROI approach, study objectives, quantitative data collection methods and the study’s data collection tools. The questions used were the generic questions from the SROI guide [11] and included:

- What has changed for you or your organization as a result of the MIYCN project activities?
- Has all the change been positive? Were some of the changes negative?
- Has anything changed that you were not expecting?
- Would the changes you have mentioned happen if the project had not been there?
- How much of a difference will each of these changes make to you or your organization?
- Was anyone else involved in making these changes happen? If so, who were they and how much change would you say was due to them?

The question guides were translated into Swahili, the most commonly-used language in the study setting. The Swahili questionnaires were not back translated but the field workers were asked to confirm if the Swahili version was the right translation of the English version. The English and Swahili guides were then reviewed by the field workers during training to confirm on the right translations to ensure that no information was missed out during the translation.

### Quantitative component.

Quantitative methods were used to assess the level of impact experienced or observed by the project participants and the frequency of participants reporting the changes in terms of nutrition, health and hygiene practices or other outcomes whether positive or negative as a result of the project. These included the perceived benefits and losses incurred as a result of the project and the estimated changes in expenditure resulting from the intervention. The questionnaires were designed by the research team under guidance from SROI experts. A pilot activity was conducted to pre-test the questionnaires, feedback from the pilot was used to review tools accordingly. Questionnaire data were collected using electronic data capture devices by a team of 10 field interviewers. The team were trained for 7 days on the SROI approach, study objectives, quantitative data collection methods and the study’s data collection tools. During the data collection process, two field supervisors were involved in supervision of the data collection process and 100% data editing to ensure high quality data.

The quantitative stakeholder survey assessed the level of impact using a Likert scale, and explored costs, duration and comparison with if the project had not taken place. The questionnaires were developed by the researchers based on the generic questions from the SROI guide [11] and were pilot tested and adjusted. For example, during the pilot the mother questionnaire had a few questions like did you participate in the project, how was it for you to participate, this was changed to “do you know about the project mentioned, did you participate and how did you find your participation”?, this was decided on after realizing that there were mothers who did not seem to know about the project. We also added a question on “who the participant had received the MIYCN information from”, this came as a result of mothers indicating that they had information and who they had received it from. This clarification was helpful in finding out if she actually participated in the project. The changes assessed included the perceived benefits and losses incurred as a result of the project and the estimated variation in expenditure resulting from the intervention. The generic questions and areas that were explored during consultation included:

- Using the Likert scale of 5 levels, please show me the level of change?
- How long do you think this change will last?
- In what other ways might the change have come about?
- What would have happened if you hadn’t been able to benefit from the MIYCN activities?

The sample of mothers was randomly obtained based on 10% in each group (intervention and control). An additional group from another study which recruited women not involved in the MIYCN study) was included to allow for comparison, as a proper counterfactual. The other stakeholders were purposefully sampled from the community. Data were collected for 281 participants, selected to represent the different stakeholders (separate questionnaire for mothers, CHVs, grandmothers, day care centers, business community, health care providers and data collection team).

### Other SROI components

Value games were used to place financial values on outcomes which did not have a market value (e.g. happier mothers). This provided a practical approach to valuing outcomes and involving stakeholders. It showed how stakeholders valued the outcomes they experienced relative to other products they also valued that have market place values (prices). Stakeholders were consulted through focus group interviews and key informant interviews. In total 16 Focus Group Discussions were conducted; 4 with mothers, 4 with fathers, 4 with grandmothers, 3 with CHWs and 1 with data collectors who were involved in the Main MIYCN study. The key informant interviews were with 3 health care providers and 3 day care center managers. The monetisation/valuation was done by carrying out a value game for each of the outcomes the participants experienced as a result of the intervention.

During value games, participants were asked to list items they also valued that have market values (prices) and to place the outcome of interest relative to these other items with market value. The average of the highest and lowest cost item was used to assess the market value of the outcome. For example, grandmothers placed the outcome ‘less burden of care’ between ‘food for one year paid’ and ‘rent for one year paid’ in terms of importance (for more information on value game, refer to [10]).

The inputs / costs that stakeholders contributed in order to make the activities possible were identified via the stakeholder questionnaire. In addition, the total intervention and research cost was estimated at using data generated by the APHRC financial system.

The SROI ratio was calculated by comparing the investments (inputs) and the financial, social and environmental returns (outcomes and impact of an intervention) based on assumptions (the assumptions are included in the Additional file 1: Table S5) as follows: SROI ratio = Total (adjusted) value of results / Total value of inputs OR SROI ratio = Total results x deadweight x attribution x inflation adjustment / Total value of inputs. Details of calculation can be found in the Additional file 1. Only final results and assumptions are presented here.

The results were reduced to recognize the influence of external influences (attribution, displacement, drop off and deadweight). Attribution was used to recognize that some of the changes were not due to the intervention only. The changes could have occurred due to other organizational influences and/or persons working together. We assessed displacement by exploring how much of the outcomes from MIYCN have displaced other outcomes that were likely to happen. Deadweight was explored in the questionnaire by asking participants to rate the likelihood of an outcome if the intervention had not taken place. Drop off was used to recognize that the effect of the outcome will decrease over time and outcomes are likely to be influenced by other factors. Finally, a discount rate was utilized to recognize that people generally prefer to receive money today rather than tomorrow which discounts the value of future benefits. Discounting was applied to the values that have duration of more than one year.

### Data analysis

#### Qualitative component

The qualitative data were analyzed through use of a thematic analysis method. A code book was developed from important areas (themes and sub themes) arising from the data and objectives of the project. The data was then coded using NVIVO qualitative software. The topics of the thematic analysis were guided and developed from the interview guides and also the recurrent and emerging information collected from the participants’ data. The pathways were identified by linking outcomes to understand the change that occurred. For example, the theory of change outcome 1.10 Healthier mother was built by creating the following pathway: Counselling on health seeking by CHWs - > Knowledge on importance of health care- > increased confidence in seeking health care- > increased child checkups- > birth at health facility- > normal birth weight- > reduced complications- > mother and baby healthy.

#### Quantitative component

The quantitative data were analyzed by use of STATA software and descriptive results were generated for each question; the frequency of a reported outcome, the average duration of a reported outcome, the average value of a reported outcome, the quantity of changes that would have occurred anyway even if the project was not implemented. In cases where comparison before and after the intervention was required, variables based on the 5-point Likert scale were regrouped into three levels of change. These levels are ‘no change’, ‘changed up the scale’ and ‘changed down the scale’. Changed up the scale is when an individual was at a lower level on the Likert scale at baseline but moved to a higher level after the intervention. The changed down the scale is the vice versa, while no change corresponds to an individual staying at the same level after the intervention as was at baseline. After deriving these three categories we computed frequencies for each category. Whether the change up or down a scale was a desirable or negative outcome depended on the particular question.

#### Other component of SROI

To estimate the cost of the intervention, assumptions were made to include 33% of the salaries of the research team based on time diaries because their time was spent partly on intervention delivery and partly on other activities such as rigorous research activities, research capacity building for the principal investigator as the original grant was for a training fellowship and academic paper writing. In addition to the APHRC costs, the mothers and grandmothers time spent during counselling sessions was estimated using what activity they could have done in the time they spent being counselled and how much income they could have made from the activity. The input value for health care providers also needed to be estimated based on the number of referrals and how much time each referral took. The time spent was then converted to how much money (salary for staff for that time/treatment). The salary costs for CHVs, and data collectors were already included in the total intervention cost.

An average of the higher and lower cost items was used to assess the market value of the outcome. For example, grandmothers placed the outcome ‘reduced burden of care’ between ‘food paid for one year costed at Ksh 365,000 for a year ’ and ‘rent paid for one year costed at 90,000 for a year’ in terms of importance. The value of reduced burden of care in terms of cost was then estimated at 365,000+ 90,000/2 = Ksh 227,500.

## Ethical approval

Ethical approval for this study was obtained from the Kenya Medical Research Institute (KEMRI) ethical review committee in June 2015. Written consent and permission to participate in the study and to record the qualitative interviews was sought from each study participant following full disclosure of the study objectives and procedures before every interview.

## Results

SROI measures the value of social benefits created by an organization, in relation to the relative cost of achieving those benefits, expressed in a SROI ratio [9–11]. The results section presents, the benefits expressed by stakeholders, the costs identified and the SROI ratio. The benefits were assessed by the combined results of the qualitative, quantitative and other component; the qualitative work identified the changes observed or experienced as a result of the intervention; the quantitative analysis assessed the level of impact experienced or observed by the project participants and the frequency of the change and the value games were used to place financial values on outcomes which did not have a market value.

### Qualitative results

The outcomes identified were in total 34 and included 3 expected (e.g. healthier children), 20 positive but not expected (e.g. mothers received more support from fathers) and 11 negative ones (e.g. increased level of worry for mothers due to challenges in introducing complementary feeding after prolonged exclusive breastfeeding) (Table 1).

**Table 1:** Outcomes grouped per outputs and stakeholders with identification of the outcomes using value based on revealed preferences, generating the most and the less social value

Stakeholders	Outcomes	Values in USD	Outcome using value based on revealed preferences approach	Outcomes that generated the most social value	Outcomes that generated the less social value
Mothers	Outcome 1.1: Increased expenditure on nutritious food and/or health care	−16,084			
	Outcome 1.2: More worried mother due to loss in baby weight and poor health	−99,181	x		x
	Outcome 1.3: Less worried mother due to better health of her children	1,378,419	x	x	
	Outcome 1.4: Decreased expenditure on food and/or healthcare	15,001			
	Outcome 1.5: Confident mother to overcome family’s pressure	1,057,745	x	x	
	Outcome 1.6: Having less burden of care	4923			
	Outcome 1.7: Improved relationship at home	1,008,474	x	x	
	Outcome 1.8: Less stressed mother because less dependent on others	349,645	x		
	Outcome 1.9: Less income due to job loss	−14,747			
	Outcome 1.10: Healthier mother	1,677,133	x	x	
	Outcome 1.11: Receiving more support from father	1682			
	Total	5,363,010			
Children	Outcome 2.1: Healthier baby	803,371	x	x	
	Outcome 2.2: Less healthy baby due to difficulty in introducing complementary feeding	−308,231	x		x
	Outcome 2.3: Better Cognitive development	839,760	x	x	
	Total	1,334,900			
Siblings	Outcome 3.1: Improved school performance for siblings	156,069	x		
	Outcome 3.2: Healthier sibling	1,101,472	x	x	
	Total	1,257,541			
Fathers	Outcome 4.1: Increased support to mother and child	0 (already valued in outcome 1.11)			
	Outcome 4.2: Increased labour participation	40,058			
	Outcome 4.3: Improved living standards at home	19,229			
	Total	59,287			
Grandmothers	Outcome 5.1: Reduced stress due to mother caring better for her children	74,294	x		
	Outcome 5.2: Happier grandmother	32,270	x		
	Outcome 5.3: Decreased healthcare expenditure	4720			
	Total	111,284			
Health care providers	Outcome 6.1: Decrease in workload due to healthier children in the community	44,279			
	Outcome 6.2: Increased workload due to mothers seeking child checkups	− 160,248			x
	Total	− 4685			
Community health volunteers	Outcome 7.1: Financial gain vs strain (salary vs own contribution to vulnerable children)	7376			
	Outcome 7.2: Increased stress due to the difficulties posed by the work	−21,859	x		
	Outcome 7.3: Increased confidence	63,890	x		
	Total	49,407			
Data collectors	Outcome 8.1: Increased income	882			
	Outcome 8.2: Increased confidence	0 (valued in outcome 10.1)			
	Outcome 8.3: Increased stress due to the difficulties posed by the work	0			
	Total	882			
Day-care centers	Outcome 9.1: Increased stress due to increased enrollment	− 3684	x		
	Outcome 9.2: Increased in expenditure due to improved hygiene and nutritious food provided	− 6703			
	Outcome 9.3: Increased attendance of children	4041	x		
	Total	− 6346			

This section focuses on the most important drivers in terms of social value to conceptualize a theory of change. Using the outcomes identified, pathways were explored to create a theory of change. The intervention contributed to the improved health of mothers and their children. Participants’ narratives are demonstrative that mothers have very well understood the MICYN, ANC/PNC and WASH messages and have changed behaviors towards optimal practices.

Mothers felt that due to use of ANC and PNC services, they faced less complications at birth and did not have to experience caesarian section deliveries. This was in comparison with the births of their other children. They knew better what to eat during pregnancy and felt stronger from it. Harmful behaviors such as use of drugs were also stopped or reduced during pregnancy resulting in normal weight babies at birth. The knowledge gained in family planning helped mothers to avoid a pregnancy soon after birth and thus to breastfeed for longer which resulted in reported better growth and health of children. Participants also reported that they exclusively breastfed for six months and extended breastfeeding to two years as counselled by CHVs in line with policy guidelines, and for some this meant stopping work to do so as their working environment was not conducive to breastfeeding. For some of the mothers, breastfeeding contributed to more relaxed babies but some also felt that introduction of complementary feeding after six months exclusive breastfeeding was challenging and meant that children lost weight during the time of introduction. For previous children born before the intervention, they were started on foods early even before they were aged one month, so they got used to food early.

Having more relaxed children who can sleep well meant having more time to carry on household chores. This time available was associated with employment of optimal practices with regards to hygiene within the household. Mothers also reported improving hygiene practices related to a child’s care. As a result, mothers recognized a decrease in diarrhea incidence and better weight gain of their child compared to their other children. Overall they felt more confident to seek appropriate health care for themselves and their children. They paid more attention to their children’s nutrition and requested improved nutrition and hygiene practices when leaving their children at the day care center.

The intervention created a more peaceful and supportive environment for child growth. Mothers were less stressed around birth and breastfeeding times because of the personalized counselling and care received. Fathers recognized the benefit of counselling towards mothers and children’s wellbeing. They were themselves happier and reported being more willing to contribute financially to the children’s needs. As children were less often sick, fathers were able to miss less days at work and thus bring more income home. Overall the relationships at home benefited from a less ‘stressed’ atmosphere.

Siblings of the children in the study also gained from this situation and received more attention from their mothers with regards to their education. Mothers had more free time resulting from healthier and happier babies and were therefore able to attend school activities. The grandmothers who were involved in looking after children, were also more relaxed and happier as the burden of care was lighter due to mothers caring for their children and healthier children.

The MIYCN intervention led to change in spending and earnings for the household. Mothers who had to stop working to exclusively breastfeed lost their income for several months and were not guaranteed to find work again. Mothers and grandmothers reported savings on health care expenditure as the child was less sick, and on maternal milk substitutes as they breastfed more. But other mothers recognized that following the MIYCN messages meant more expenditure towards health care (due to increased referrals by CHVs) and nutritious food resulting from advice by the CHVs. Fathers were able to work more and hence earned more and even reported saving money to start businesses, as they saved time otherwise used on health care for the child.

The MIYCN intervention put pressure on day care centers and health care facilities. Day care centers had to meet the requirement of mothers for better hygiene and food without being able to increase fees. These increased expenditures were not compensated for by the increased income due to higher attendance of children (children were reported to miss less days due to illnesses). In the short run, health care centers faced increased referral of malnourished and sick children and of children for checkups from the community by CHVs, which posed a heavier workload on health staff.. Nevertheless, in the longer run, they expected a decrease in work load as children in the community were healthier.

The MIYCN intervention built confidence and skills for field staff but with increased stress. Field staff including data collectors and CHVs felt better skilled due to the training received and the working experience. These placed them in a better social situation within the community and meant that their chances for future employment increased. CHVs felt empowered especially in being the link between the community and the health care services. On the other hand, they faced a highly stressed situation in the communities being confronted by extreme poverty and vulnerabilities without the adequate support .

### Quantitative results

#### Costs / Inputs

The inputs were what stakeholders contributed in order to make the activities possible. The total intervention and research cost was estimated at USD$ 394,544 (Table 2).

**Table 2:** APHRC intervention and research costs for MIYCN intervention

Period of Report: March 01, 2012 - October 31, 2014		
	Cumulative Expenditure	Cumulative Expenditure
Cost item	To Date (Ksh)	To Date (US$)
Salaries and consultant fees	19,994,314	231,936
Training workshop, Meetings, Workshops, travel and accommodation	2,333,722	27,000
Telephone, email, internet and bank charges,	400,884	4661
Printing & Stationery	640,300	7492
Motor Vehicle Running Costs	1,636,534	18,934
Non-Capital Office Equip & Tools and others	478,641	5531
Training Expenses	2,017,102	23,600
Other Program Expenses	243,145	2852
Field Office Expense	6,232,483	72,539
TOTAL	33,977,127	394,544

In addition to the APHRC costs, the mothers and grandmothers time spent during counselling sessions was estimated to be up to USD$3087. The input value for health care providers estimated based on the number of referrals and how much time each referral took was USD$21,180. The total input was USD$419,716.

#### Benefits

The SROI ratio was calculated using values placed by stakeholders on the outcomes identified by themselves. The outcomes that did not have market value (Table 1) were valued by the stakeholders themselves through value games.

The total social value created by the project was USD$ 8 million (this is the total for all stakeholders: mothers, children, siblings, fathers, grandmothers, health care providers, CHVs, data collection team, day care centers). The Total Present Value for the project for 5 years, at a discount rate of 6.5%, was USD$ 29.8 m. The Net Present Value was USD$ 21.7 m (the Total Present Value minus the total of all inputs) (Table 3). Thus, the SROI ratio was of USD$ 29.4 m / 0.4 m (Net Present Value / Input) = USD$ 71: USD$ 1. This means for every dollar of investment in the MIYCN project, USD$ 71 of social value was created.

**Fig. 1:**
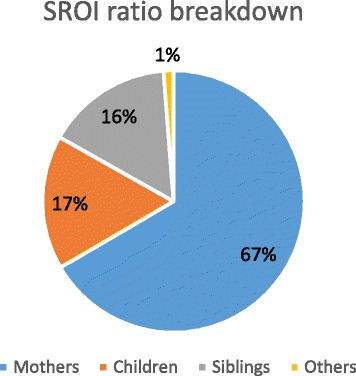
SROI ratio breakdown per stakeholder group

Mothers, children and siblings were the stakeholder groups that benefited the most with mothers representing 67%, children 17% and siblings 16% of the impact (Fig. 1).

The other stakeholders, including grandmothers, fathers, health care centers, and day care centers benefited from 1% of the social value generated. All stakeholder groups had a positive impact except for health care provider and day care centers (Table 3).

**Table 3:** Summary findings of impact by stakeholder group in USD (discount rate: 6.5%)

	Total impact	Year 0	Year 1	Year 2	Year 3	Year 4	Year 5	Total Present value	Net Present Value
Mothers	5,363,010	5,363,010	5,035,690	3,797,333	2,176,684	1,636,006	1,227,893	19,236,616	19,233,529
Children	1,334,900	1,334,900	1,253,428	941,542	707,261	531,276	399,081	5,167,488	5,167,488
Siblings	1,257,541	1,257,541	1,180,790	886,978	666,275	500,488	375,954	4,868,026	4,868,026
Fathers	59,287	59,287	55,669	41,817	31,412	23,596	17,725	229,506	229,506
Grandmothers	111,284	111,284	104,492	78,492	58,961	44,290	33,270	430,789	429,884
Healthcare providers	− 115,969	−115,969	− 108,891	−81,796	23,460	17,623	13,238	− 252,336	− 273,516
CHVs	49,407	49,407	46,392	34,848	33,850	25,427	19,100	209,025	209,025
Data collection team	857	857	804	604	467	351	264	3347	3347
Day care	−6346	−6346	− 5958	− 4476	− 1410	− 1059	1208	−18,042	−18,042
*APHRC*									394,544
Total	8,053,972							29,874,419	29,454,703
SROI ratio per amount invested	71								

The outcomes that generated the most social value to mothers and children were ‘healthier mother’ and ‘less worried mother due to better health’ (Table 1).

The most important negative outcomes (with the most value lost) were ‘less healthy baby due to difficulty in introducing complementary feeding after exclusive breastfeeding’ and ‘Increased workload for health workers due to more referrals by CHVs and mothers seeking child checkups’ (Table 1).

#### Sensitivity analysis

Sensitivity analysis was used to test the variables and assumptions used based on base and new scenarios. We checked changes for estimates of deadweight, attribution, displacement, drop-off and discount rate, the frequency of the outcome and the value of outcomes, where we used value games. The sensitivity analysis showed that the ratio (71) can fluctuate from 34 to 136 depending on new case values (Table 4). The SROI ratio is most sensitive to variation in value of outcomes that were based on value game exercises, deadweight and frequency used in key outcomes.

**Table 4:** Base and new case scenarios

Sensitivity analysis	Base case	New case	New ratio
Attribution	0–25%	50%	USD$ 44:1
Deadweight	5 – 100%	50%	USD$ 47:1
Displacement	No displacement	25%	USD$ 51:1
Drop-off	20%	50%	USD$50:1
Discount rate	6.50%	3.30%	USD$72:1
Outcome frequency use			
Healthier baby	99%	50%	USD$56:1
Healthier sibling	59%	50%	
Outcome frequency use			
Healthier baby	99%	50%	USD$47:1
Healthier sibling	59%	0%	
Outcome frequency use			
Healthier mother	97%	48.50%	USD$60:1
Value of the outcome using value game	On average USD$ 2150	Value divided by 2	USD$34:1
Value of the outcome using value game	On average USD$ 2150	Value multiplied by 2	USD$136:1

## Discussion

This paper presents the social value of a community-based nutrition intervention aimed at counselling and supporting mothers for optimal infant and young children feeding practices. Results showed that the intervention created a theory of change in the community regarding children and mother’s improved health and generated an important social value, mostly towards mothers and children, who were the primary beneficiaries.

A comparison of the SROI ratio with other interventions in health and nutrition shows that this MIYCN intervention had the largest ratio [12]. In Banke-Thomas et al. (2015) [12] systematic review on SROI, 12 studies were identified in health promotion [13–24], four in child health [25–28] and three in nutrition [15, 29, 30]. The reported SROI ratios varied from 1.1:1 to 65:1. There were two studies from Kenya that used SROI, one on reproductive health and one on water interventions [31, 32] with lower SROI ratios of respectively, 1:25 and 1:8. The fact that the SROI ratio calculated in our study was the highest may be due to the lack of comparison basis as SROI is still a relatively new method. It is also possible that SROI was used in other nutrition promotion interventions but that the findings were not published in peer reviewed journals as so far most the studies using SROI arein grey literature.

The findings here add evidence to previous results on effectiveness and impact of preventive nutrition programs on children’s nutritional status in LMICs as presented in the Lancet series on Maternal and Child Undernutrition [6]. The series showed that there was extremely limited evidence of breastfeeding promotion impacting on children’s nutritional status; the limited number of studies that assessed nutritional status did not show an impact on weight nor length of infants [6]. The findings here were able to confirm the potential effectiveness of MIYCN reported in Kimani-Murage et al. (2016) [8] on optimal infant feeding practices and improved child health. Furthermore, this study has added to previous findings by creating a theory of change that explains how the intervention activities led to these positive outcomes but also to negative ones. An important finding was that the intervention created a favorable environment towards child’s health where improved MICYN, WASH and family planning practices were employed and where mothers, fathers and grandmothers were less stressed about the child’s health. This is an environment where a couple agrees for the mother to stop working in order to exclusively breastfeed their baby as her working environment was not conducive to do so; or where fathers were willing to provide additional financial support towards child’s growth. This supportive environment leading to confident, less worried and less stressed mothers is recognized to be extremely important to successfully breastfeed children [33]. The intervention also reduced the burden of care for the grandmothers, who were often the caregivers of children born to young mothers. Previous research found that child care by young mothers was compromised due to limited knowledge and competence of the young mothers on child care, leading to dependence on their own mothers and mothers-in-law (grandmothers of the children), and also lack of commitment [34]. Lower burden of child care on the grandmothers freed their time for labor force participation. Interventions that aim to counsel on breastfeeding exist [6] but the MIYCN study was slightly different in the design; the timing was different as counselling started when women were pregnant and the topics of counselling were not focused only on MIYCN but included ANC/PNC, WASH and family planning. Findings showed that these differences were important and contributed to positive outcomes for maternal and child health and wellbeing. The children were shown by this method to have less SROI than mothers despite the intervention being targeted at them. It could be hypothesized that because they were too young to be given their own voice in this type of evaluation (mothers had to report on both their own and their children’s benefits). Other SROI assessments did include children as stakeholders (4 studies in child health) but the children were older [26–28] and were able to be interviewed or the study was evaluative and did not interview children [25]. Nevertheless, it is interesting to note that in Biswas et al.’ study (2010) [26], parents/caregivers benefited significantly more than the children in terms of value created. Whilst this appeared to be an unexpected result as in the MIYCN program children were the primary beneficiaries, it was due to the high financial value generated by tangible improvements in livelihood status through income-generating or increased wage earning opportunity for their parents. The authors noted that the benefits of improved parent/caregiver income would positively impact children. Similarly, here, it is expected that the social value gained by parents will benefit the children as identified already in the theory of change; more confident mothers can use optimal IYCF practices. Additionally, benefits to child health and development are long-term, and may not have been fully captured in our assessment.

The social value generated by the intervention brings new evidence on the potential impact of the proposed BFCI in Kenya. This is key information as the existing evidence so far is extremely limited on effectiveness of such a program [8]. In addition, this is the first study exploring impact from the stakeholder point of view from their personal experience, which resulted in their own evaluation of the intervention. This is useful in scaling up the intervention with regard to the proposed national BFCI program in Kenya as policy engagement has already been established through the pilot design and the involvement of the national MIYCN steering committee formed by representatives of Ministry of health (Unit of Nutrition & Dietetics), UNICEF and relevant NGOs. While the intervention led mostly to positive outcomes, negative outcomes were also identified. The fact that negative outcomes were identified and presented is demonstrative of the approach taken of not limiting the assessment to ‘positive outcomes’ only. This is new knowledge as the Lancet systematic review of current evidence on maternal and child undernutrition did not include a review of the negative outcomes [6]. We suggest here some programmatic recommendations to minimize these negative outcomes in future BFCI programs in Kenya (Table 5).

**Table 5:** Programmatic recommendations to minimize negative outcomes in future BFCI programs

Stakeholder	Outcome	Recommendations
Mothers	Outcome 1.1: Increased expenditure on nutritious food and/or health care	Provide social protection measures or empowerment for food and healthcare.
	Outcome 1.2: More worried mother due to loss in baby weight and poor health	Provide targeted counselling around complementary feeding introduction.
	Outcome 1.9: Less income due to job loss	Provide small subsidy such as cash transfer or empowerment of women for baby friendly income generating activities. Advocate for maternity leave benefits.
Children	Outcome 2.2: Less healthy baby due to difficulty in introducing complementary feeding	Provide targeted counselling around complementary feeding introduction.
Healthcare providers	Outcome 6.2: Increased workload due to increased referrals and mothers seeking child checkups	Provide extra staff and support via Ministry of Health.
CHVs	Outcome 7.2: Increased stress due to the difficulties posed by the work	Provide support to vulnerable mothers and children via social protection measure or financial empowerment. Provide psycho social support to CHVs.
Data collectors	Outcome 8.4: Financial strain due to financial aid given to vulnerable mothers	Provide support to vulnerable mothers and children via social protection measure or financial empowerment. Provide psycho social support to data collectors.
Day care centers	Outcome 9.1: Increased stress due to increased enrollment without increased financial gains	Provide small subsidy for food and hygiene or microfinance services.
	Outcome 9.2: Increased in expenditure due to improved hygiene and nutritious food provided	Provide small subsidy for food and hygiene or microfinance services.

The knowledge shared here from applying SROI to a nutrition intervention with scientific rigor and research expertise could be useful material for future use throughout the nutrition sector, and can provide practical reference materials to other SROI practitioners implementing such studies in developing country contexts.

The recommendations for future programming or scale up were for National and County Governments to roll out the BFCI program as a health promotion tool, and to support the community health strategy through funding CHVs as an essential component of the BFCI program. BFCI has positive impacts on mother and child health as well as the health and wellbeing of other family and community members including fathers and grandmothers. Incentives for community health volunteers to motivate them should be provided combined with adequately training them on handling psychosocial issues. The health facilities in the community should be reinforced to face the change in workload and increased referrals. Day care centers are places where children’s nutrition and growth could be put at risk. Subsidies or contribution towards ‘child friendly’ day care centers that promote child health would likely result in positive changes for children. The community should be empowered economically through social protection measures and empowerment programs such as job creation and support of mothers who wish to successfully combine work with breastfeeding as some mothers had to stop working to breastfeed. Men (fathers) should be included in BFCI interventions as they contribute to its success and positive outcomes by supporting their wives and children financially and beyond. Finally, for researchers, NGOs, and donors the SROI approach should be more widely used in evaluation of interventions in order to identify negative outcomes and value social outcomes.

### Limitations

The limitations were related to the complexity of assessing future health benefits of a current intervention and the challenges in valuing non-market valued outcomes. We decided therefore not to value future health benefits and to limit the duration of impact to not more than 5 years. We feel that this may underestimate the social value of the intervention but without data on how to evaluate these, we preferred not to include them. We used willingness to pay via value games to monetize outcomes without market value such as confidence, burden of care, worry, happiness etc. While value games exercises were done to minimize subjectivity and to reach a consensus per stakeholder group, sensitivity analysis showed that the SROI ratio was mostly sensitive to these. Although in Banke-Thomas (2015) [11], there is an agreement that primary beneficiaries are best placed to identify and value the outcomes themselves, it was suggested in order to make the process more robust, that the financial proxies described by beneficiaries should be tested through further research for appropriateness and relevance. A proxy verification process could be integrated into monitoring and evaluation procedures to ensure that financial proxies reflect current trends and perceptions of beneficiaries.

## Conclusion

The MIYCN intervention showed an important social value with mothers and children benefiting the most. Mothers and children reported better health, as well as increased confidence of mothers. Overall, the intervention had a negative social value on day care centers and health care providers putting too much pressure on them without providing extra support. These findings can inform the design of future programming by maximizing the social value the intervention created towards improving health of mothers and children, and by targeting resources to manage negative outcomes towards day care centers and health care providers. The policy recommendations from this work are to roll out the BFCI program as a priority health promotion tool, as it is likely to have huge health and social benefits, and to support the community health strategy through funding CHVs as an important component of the BFCI program. When implementing the BFCI, it is important to recognize that there is a need to ensure that adequate support for health service and day care provision is available for the initiative to have a greater chance of success in creating social outcomes.

## Additional file

Additional file 1: **Table S1.**Steps in the proposed Baby Friendly Community Initiative (BFCI) program in Kenya, Step Description, **Table S2.** Content of counselling messages, **Table S4.**List of stakeholders, **Table S5.** Assumptions for base case scenario variables. (DOCX 18 kb)

## Abbreviations

APHRC: African Population and Health Research Center
BFCI: Baby Friendly Community Initiative;
BFHI: Baby-friendly Hospital Initiative;
CHV: Community Health Volunteer;
IYCF: Infant and young child feeding;
MIYCN: Maternal, infant and young child nutrition

## References

[1] Kenya National Bureau of Statistics, Ministry of Health, National AIDS Control Council, Kenya Medical Research Institute, National Council for Population and Development: Kenya Demographic and Health Survey 2014: Key indicators report. In Nairobi; 2015.

[2] Kimani-Murage EW (2011). Patterns and determinants of breastfeeding and complementary feeding practices in urban informal settlements, Nairobi Kenya. BMC Public Health.

[3] Concern (2014) ProPAN ASSESSMENT REPORT-VIWANDANI SLUMS: NAIROBI-KENYA. Concern Worldwide.

[4] Kimani-Murage EW, Muthuri SK, Oti SO (2015). Evidence of a double burden of malnutrition in urban poor settings in Nairobi, Kenya. PLoS One.

[5] Olack B, Burke H, Cosmas L (2011). Nutritional status of under-five children living in an informal urban settlement in Nairobi, Kenya. J Health Popul Nutr.

[6] Bhutta ZA, Das JK, Rizvi A, Gaffey MF (2013). Evidence-based interventions for improvement of maternal and child nutrition: what can be done and at what cost?. Lancet.

[7] WHO. Baby-friendly Hospital Initiative. Website accessed: http://www.who.int/nutrition/topics/bfhi/en/ [01.05.2016].

[8] Kimani-Murage EW, Norris SA, Mutua MK, Wekesah FM, Wanjohi M, Muhia N, Muriuki P, Egondi T, Kyobutungi C, Ezeh AC, et al. Potential effectiveness of community health strategy to promote exclusive breastfeeding in urban poor settings in Nairobi, Kenya: a quasi-experimental study. J Dev Orig Health Dis. 2016; 10.1017/S2040174415007941 26708714

[9] Browers et al. SROI: a practical guide for the development cooperation sector. Website http://bigpushforward.net/wp-content/uploads/2011/09/sroi_practical_guide_context_international_cooperation.pdf. 2010.

[10] Goudet S, Wainaina C, Macharia T, Wanjohi M, Wekesah F, Muriuki P, Samburu B, Griffiths P, Kimani-Murage E (2016). Return on investment (SROI) assessment of a baby-friendly community initiative in urban poor settings, Nairobi, Kenya. Field exchange.

[11] SROI network. A guide to Social Return on Investment. Website http://www.thesroinetwork.org/sroi-analysis/the-sroi-guide. 2012.

[12] Banke-Thomas, A. O., Madaj, B., Charles, A., & van den Broek, N. Social return on investment (SROI) methodology to account for value for money of public health interventions: a systematic review. BMC Public Health 2015, 15, 582. 10.1186/s12889-015-1935-7 10.1186/s12889-015-1935-7 PMC4477315 26099274

[13] Lukoseviciute L (2010). Social return on investments in smoking cessation policy in the Netherlands.

[14] Jones M (2012). The social value of a community-based health project: healthy living Wessex social return on investment report. Project report.

[15] Bradly J, Bolas C (2013). Social return on investment (SROI) of substance misuse work Leicestershire youth offending service.

[16] Carrick K, Lindhof J (2011). The value of walking: a social return on investment report of a walking project.

[17] Carrick K. Glasgow health walks social return on investment analysis: 1st April 2011 to 31st. Alloa: Paths for All. March 2012:2013.

[18] Age Concern Kingston upon Thames (2012). Stay well at home social return on investment (SROI) evaluation report.

[19] Pank H. Gorgie City farm community gardening project: social return on investment (SROI) report.Edinburgh: Federation of City Farms & Community Gardens Gardening Project; 2011.

[20] Lobley N, Carrick K, McNiven V (2012). “Bums off seats” a social return on investment evaluation report.

[21] Kennedy R, Phillips J. Social return on investment (SROI): a case study with an expert patient Programme. SelfCare. 2011;2:10–20. http://selfcarejournal.com/article/social-return-on-investment-sroi-a-case-study-with-an-expert-patient-programme/

[22] Shipley R, Hamilton L (2011). Healthwise hull.

[23] Social Ventures Australia (SVA) Consulting (2013). Spinal cord injuries Australia’s National Walk on Program.

[24] Satchwell Smith P (2012). Social return on investment analysis. Harare: positive initiative trust (PIT).

[25] Bhaumik U, Norris K, Charron G, Walker SP, Sommer SJ, Chan E (2013). A cost analysis for a community-based case management intervention program for pediatric asthma. J Asthma.

[26] Biswas K, Kummarikunta G, Biswas A, Tong L (2010). Social return on investment: Chaha program.

[27] Ltd FEW-K (2011). Social return on Investment for Whizz-Kidz’ services: an evaluation.

[28] Misje T (2011). Social return on investment (SROI): SROI analysis of health services delivered on the streets of Moshi, Tanzania.

[29] McCorriston E (2012). SROI analysis: Hertfordshire community Meal’s meals on wheels service.

[30] Varua M, Stenberg L. Social Return on Investment: A Case Study of a Community NGO in Sydney. 2009 Sustainability research node symposium, Sustainability: Dimensions and Intersections. 2009.http://researchonline.nd.edu.au/bus_conference/22/

[31] Jönsson J, Wikman A, Wätthammar T. Social return on investment, SROI, the value added for families before and after using Solvatten in the Bungoma district in western Kenya. Uppsala: Swedish university of. Agric Sci. 2011;

[32] Aid C. Filling the gaps: a social return on investment analysis. London: nef consulting. 2013;

[33] Richter L (2015). The importance of caregiver-child interactions for the survival and healthy development of young children: a review.

[34] Kimani-Murage EW, Wekesah F, Wanjohi M, Kyobutungi C, Ezeh AC, Musoke RN, Norris SA, Madise NJ, Griffiths P. Factors affecting actualisation of the WHO breastfeeding recommendations in urban poor settings in Kenya. Matern Child Nutr. 2014; 10.1111/mcn.12161 PMC6860346 25521041

