# Supplementary material for: Social value of a nutritional counselling and support program for breastfeeding in urban poor settings, Nairobi
Source: BMC Public Health. 2018 Apr 2;18:424. doi: 10.1186/s12889-018-5334-8 (PMC5880085; doi:10.1186/s12889-018-5334-8)
Supplement: Supplementary file 1 — Table S1.Steps in the proposed Baby Friendly Community Initiative (BFCI) program in Kenya, Step Description, Table S2. Content of counselling messages, Table S4.List of stakeholders, Table S5. Assumptions for base case scenario variables. (DOCX 18 kb) [file 12889_2018_5334_MOESM1_ESM.docx]

**Table 1**: Steps in the proposed Baby Friendly Community Initiative (BFCI) program in Kenya

Step Description

Step 1 Have a written MIYCN policy summary statement that is routinely communicated to all health providers, community health volunteers and community

Step 2 Train all health care providers and community health volunteers in the knowledge and skills necessary to implement the MIYCN policy

Step 3 Promote optimal maternal nutrition among women and their families

Step 4 Inform all mothers and their families about the benefits of breastfeeding and risks of artificial feeding

Step 5 Support mothers to initiate breastfeeding within the first hour of birth, establish and maintain exclusive breastfeeding for first 6 months

Step 6 Encourage sustained breastfeeding beyond 6 months to 2 years or more alongside timely introduction of appropriate, adequate and safe complementary foods

Step 7 Provide a welcoming and conducive environment for breastfeeding families

Step 8 Promote collaboration between health care staff, maternal, infant and young child nutrition support groups and the local community MIYCN maternal, infant and young child nutrition

**Table 2:** Content of counselling messages

| **Maternal Nutrition:**   - Food portions during pregnancy and lactation - Appropriate foods (nutritious, affordable, and locally available) during pregnancy and lactation - Frequency of feeding during pregnancy and lactation |
| --- |
| **Breastfeeding:**   - Breast positioning and attachment - Immediate initiation of breastfeeding after birth - Exclusive breastfeeding for six months - Frequency and duration of breastfeeding - Expressing breast milk, storage and cup feeding - Dealing with breast conditions - Breastfeeding for HIV positive women |
| **Complementary feeding:**   - Timely initiation of complementary foods - Appropriate complementary foods (nutritious, affordable, and locally available) - Feeding frequency and quantity - Appropriate feeding practices including hygiene and responsive feeding behaviors - Safe preparation and storage of foods |

**Table 4: List of stakeholders**

| **Stakeholders** | **Explanations** |
| --- | --- |
| Mothers in the two slums involved in the intervention | They were the primary beneficiaries of the intervention. Mothers were counselled and followed up by community health volunteers (CHVs) from pregnancy to when the child was over six months. |
| Children in the intervention | They were the primary beneficiaries who should have benefited from better nutrition and WASH practices as their mothers were counselled on optimal practices in nutrition, health and WASH. |
| Fathers of children involved in the intervention | Some of the fathers attended the counselling sessions. Their wives shared knowledge with them. |
| Grandmothers of children involved in the intervention | They often looked after their grand-children in the absence of the mother and attended some of the counselling sessions. Their daughters could have shared knowledge with them. |
| Health care providers in the community | They could have been consulted as mothers were sensitized towards the importance of seeking health care. |
| Community health volunteers | CHVs were trained to counsel mothers. They could have gained knowledge and skills through training and experience. They were also given a monthly incentive. |
| Data Collection team | The data collection team was trained and could have benefited from the knowledge gained. They were also paid a salary. |
| Day care center managers | They could have benefited from the intervention as counselled mothers could have asked for better nutrition and hygiene practices in day care centers. |

**Table 5: Assumptions for base case scenario variables**

|  | **Base case** | **Source** |
| --- | --- | --- |
| **Exchange rate Kenya Shilling to US dollar** | 0.00098 | Exchange rate March 2016 |
| **Frequency of the outcomes identified^[[1]](#footnote-1)^** | 4 - 100% (mean: 67%) | Stakeholder questionnaire. Frequency of a reported outcome |
| **Sample size** | Mothers, children, siblings: 1100, Fathers: 935, Grandmothers: 52, Healthcare provider: 30; Data collection team: 15; Day care center: 10 | RCT data for mothers, children. Extrapolation based on stakeholder questionnaire and the rate of participation. |
| **Quantity** | Min:2; Max:1100; Mean: 397 | Based on frequency * sample size |
| **Duration** | 1 - 5 years (mean: 4.11) | Stakeholder questionnaire. Duration of a reported outcome. |
| **Value of all outcomes** | mean: USD$420 | Based on cost estimate in stakeholder questionnaire, willingness to pay for outcomes that do not have market value |
| **Value of the outcome using value games only^[[2]](#footnote-2)^** | mean: USD$2150 | Based on willingness to pay for outcomes that do not have market value: table 7 |
| **Attribution** | 0 – 25% | Based on qualitative data to estimate the attribution rate. When value was negative, no attribution rate was used. |
| **Deadweight** | 5      – 100% | Based on stakeholder questionnaire and the likelihood of an outcome if the intervention had not taken place. When we did not have frequency, we used 5% to guard against the possibility of over claiming. |
| **Displacement** | No displacement | No evidence for displacement. |
| **Drop-off** | 20% | Based on the assumption that the effect of the intervention will be 0 after 5 years. |
| **Discount rate** | 6.50% | Based on Kenya inflation rate |

1. The outcomes were identified in the ‘mapping outcomes’ phase. Outcomes can be positive or negative and represent the material results of an intervention. [↑](#footnote-ref-1)
2. Value of the outcomes using value games only is included as a different method (willingness to pay) was used to put a monetary value. [↑](#footnote-ref-2)
